# Clinicopathological and prognostic significance of COX-2 immunohistochemical expression in breast cancer: a meta-analysis

**DOI:** 10.18632/oncotarget.13990

**Published:** 2016-12-16

**Authors:** Feng Xu, Mengxin Li, Chao Zhang, Jianxiu Cui, Jun Liu, Jie Li, Hongchuan Jiang

**Affiliations:** ^1^ Department of Breast Surgery, Beijing Chao-Yang Hospital, Capital Medical University, Beijing, China, 100020

**Keywords:** breast cancer, COX-2, prognosis, meta-analysis

## Abstract

The prognostic significance of COX-2 in patients with breast cancer remains controversial. The aims of our meta-analysis are to evaluate its association with clinicopathological characteristics and prognostic value in patients with breast cancer. PubMed, EMBASE, Web of Science, the Ovid Database and Grey literature were systematically searched up to May 2016. Twenty-one studies including 6739 patients with breast cancer were analyzed. The meta-analysis indicated that the incidence difference of COX-2 expression was significant when comparing the lymph node positive group to negative group (OR = 1.76, 95% CI [1.30, 2.39]) and the tumor size ≥ 2cm group to the tumor size < 2cm group (OR = 1.71, 95% CI [1.22, 2.39]). None of other clinicopathological parameters such as the ER status, PR status, HER2 status and the vascular invasion status were associated with COX-2 overexpression. The detection of COX-2 was significantly correlated with the disease-free survival (DFS) of patients (HR = 1.58, 95% CI [1.23, 2.03]) and the overall survival (OS) of patients (HR = 1.51, 95% CI [1.31, 1.72]). Our meta-analysis demonstrates that the presence of high levels of COX-2 is associated with poor prognosis for breast cancer patients and predicts bigger tumor size and lymph node metastasis.

## INTRODUCTION

As one of the most frequently diagnosed malignant tumor, breast cancer (BC) ranks first among female cancer deaths. In 2015, for example, approximately 234,190 new cases in the USA diagnosed with breast cancer annually, with an estimated 40,730 deaths [1]. Despite the development of surgery and adjuvant chemotherapy has significantly improved the clinical survival for BC patients over past few years, the occurrence of breast cancer is still on the rise. The prognostic factors that have been implicated include human epidermal growth factor receptor (HER2), estrogen/ progesterone receptor (ER/PR), tumor size, lymph nodes metastasis, and response to chemotherapy [2]. However, the mechanism of the clinical outcome in breast cancer patients has yet to be completely understood. Therefore, novel prognostic biomarkers and therapeutic targets are needed to be identified for the management of breast carcinoma.

Recently, research on cyclooxygenase (COX) in tumorigenesis and tumor progression has become a hotspot [3]. Apart from the newly discovered COX-3 isoform, COX-1 and COX-2 are involved in the prostanoids synthesis [4]. These isoenzymes locate in different chromosomes and differ considerably in patterns of expression and biology. Among them, COX-2 is usually undetectable in normal tissues. Various stimulating factors such as hormones, cytokines, and dysregulated oncogenes have all been shown to cause induction of COX-2 expression [5]. It is reported that COX-2 have an carcinogenic impact on various cancers, such as lung [6], oesophagus [7], breast, colorectal [8] and pancreas cancer [9].

In breast cancer, increased COX-2 expression is found in cancerous tissue compared to the corresponding *paracancerous tissues* [10]. Many researches have evaluated the association between COX-2 overexpression and the prognosis of breast cancer patients. However, the findings with respect to COX-2 expression in BC specimens are varying and sometimes conflicting. In order to clarify the question, we collected all eligible articles to determine the association between COX-2 overexpression and clinicopathological parameters/prognoses in BC patients.

## RESULTS

### Study selection and characteristics

**Figure 1:**
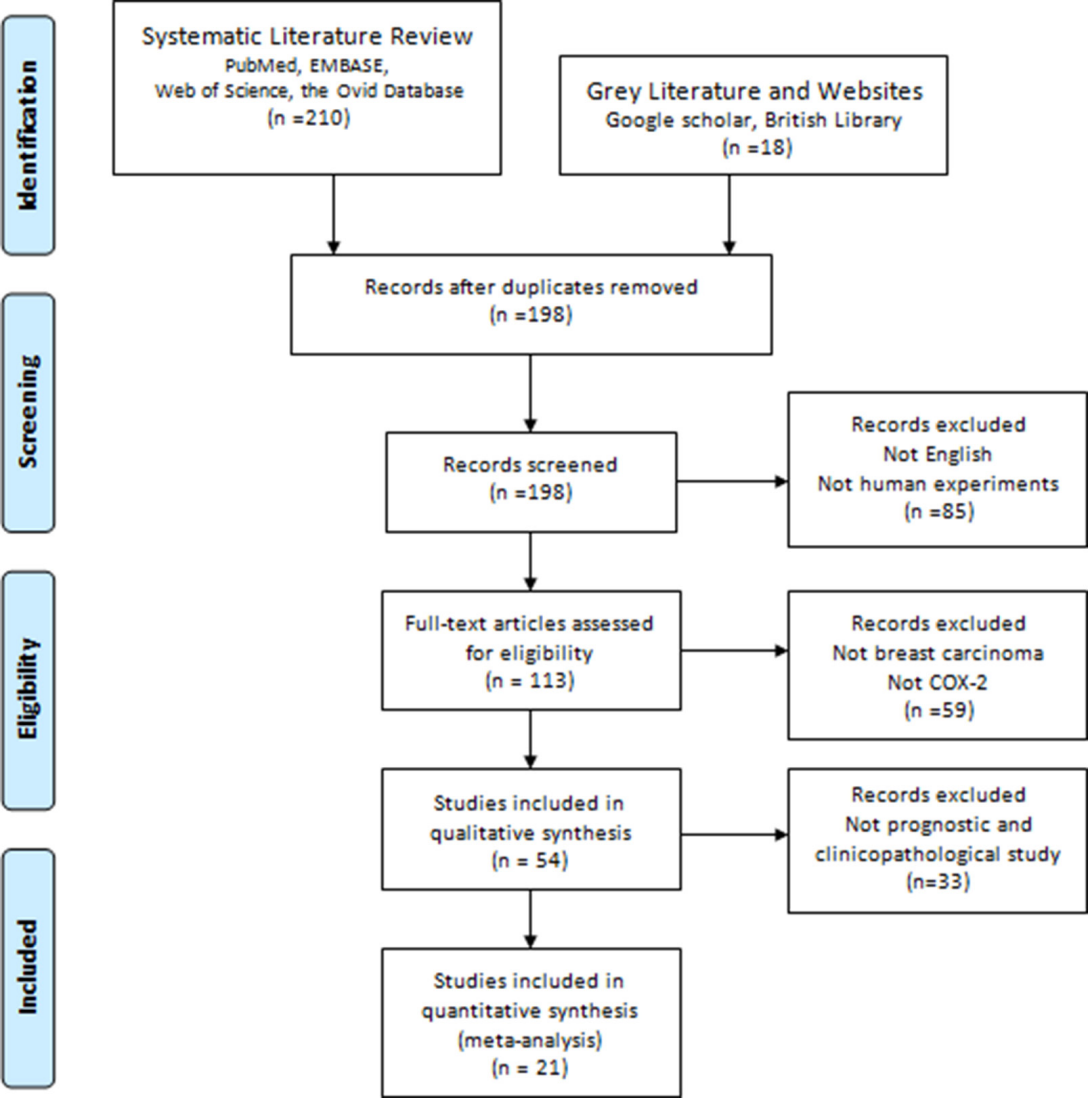
PRISMA flow chart of the literature search

228 relevant manuscripts were initially retrieved. After using the search strategy mentioned above, a total of 21 studies [11–31] comprising 6739 patients were considered in this meta-analysis (Figure 1). The major features and quality assessment of the 21 eligible articles were summarized in Tables 1 and 3. The studies were conducted in 15 countries (China, Finland, Korea, Portugal, Austria, Poland, Sweden, Germany, Italy, Turkey, Brazil, Turkey, Tunisia, Norway, and the United States). Fourteen studies were performed using immunohistochemistry (IHC) method, and the remaining seven studies followed tissue microarray (TMA) method. Eleven studies evaluated the prognostic effect of COX-2 overexpression in BC patients. Among them, ten studies reported the overall survival(OS) of BC patients, and six for disease-free survival(DFS). The occurrence of COX-2 overexpression in each study ranged from 27.9% to 81.4%. The cut-off values of IHC/TMA evaluation were inconsistent. Regarding different anti-COX-2 monoclonal antibodies, ten studies used clone 160112 from Cayman company, and eleven focused on others. We extracted hazard ratios and their corresponding 95% CIs from the graphical survival curve in 5 univariable analyses and reported them directly in 6 multivariate analyses. Moreover, none of the patients received neo-adjuvant chemotherapy prior to surgery.

**Table 1:** Main characteristics and results of the enrolled studies

Authors [ref]	Year	Number of patients	Country	Detection Method	Cut-off (positive/High expression)	Antibody For Cox-2 staining	Analysis method	HR For Survival (95% CI)	Follow up (months)	Quality assessment (NOS)
Costa C [11]	2002	46	Portugal	IHC	NA (8)	NA	Univariable	Survival curve DFS:4.22 (0.26–68.49)	> 25	7
Ristimaki A [12]	2002	1576	Finland	TMA	score > 1 (592)	Clone 160112, Cayman	NA	NA	NA	8
Denkert C [13]	2003	221	Germany	IHC	Score > 6 (80)	Clone 160112, Cayman	Mutivariable	Reported DFS: 1.90 (1.00–3.61) OS:1.14 (0.67–1.93)	NA	8
Spizzo G [14]	2003	212	Austria	IHC	Score > 4 (103)	Clone 160112, Cayman	Univariable	Survival curve DFS:1.28 (0.79–2.07) OS:1.46 (0.90–2.38)	126	8
Wulfing P [15]	2003	192	Germany	TMA	score > 1 (78)	Clone 160112, Cayman	Univariable	Survival curve OS:1.56 (0.87–2.78) DFS:1.26 (0.73–2.17)	71(0–110)	7
John K [16]	2004	23	America	IHC	≥75% (15)	Clone ALX-804-112-C050, Alexis Biochemicals	Univariable	Survival curve OS:2.29 (0.30–17.48) DFS:2.08 (0.31–13.96)	48	6
Surowiak P [17]	2005	102	Poland	IHC	> 10% (46)	Clone 160112, Cayman	Univariable	Survival curve OS:3.81 (1.06–13.69)	81	8
Gunnarsson C [18]	2006	284	Sweden	TMA	NA (234)	Clone 160112, Cayman	NA	NA	NA	6
Park K [19]	2006	178	Korea	TMA	> 80% (70)	Clone 160112, Cayman	Mutivariable	Reported DFS:1.91 (1.24–2.94) OS:1.73(1.16–2.58)	42 (1–60)	7
Narssar A [20]	2007	43	America	TMA	Score > 2 (35)	Clone 160112, Cayman	NA	NA	NA	5
Zerkowski MP [21]	2007	669	America	TMA	score > 19.3 (294)	Clone 160112, Cayman	Mutivariable	Reported OS:1.66 (1.12–2.46)	106(2–636)	6
Zhang XH [22]	2008	70	China	IHC	> 5% (46)	Zymed	NA	NA	NA	7
Glynn SA [23]	2010	238	America	IHC	Score > 3 (90)	Clone 33, BD	Mutivariable	Reported OS:1.82 (1.07–3.10)	68 (12–166)	8
Miglietta A [24]	2010	91	Italy	IHC	Score > 4 (64)	Cayman	NA	NA	NA	7
Rozenowicz RD [25]	2010	41	Brazil	IHC	Score > 1 (23)	3362–100, Biovision	NA	NA	NA	6
Barisik NO [26]	2011	62	Turkey	IHC	Score ≥ 4 (47)	Clone SP392, DAKO	NA	NA	NA	6
Ciris IM [27]	2011	51	Turkey	IHC	Score ≥ 2 (30)	Clone SP21, Labvision	NA	NA	NA	7
Holmes MD [28]	2011	2001	America	TMA	Score≥1 (560)	Clone SP21, Labvision	Mutivariable	Reported OS:1.37 (1.13–1.67)	> 240	8
Sondes KC [29]	2011	83	Tunisia	IHC	Score ≥ 4 (46)	Clone M-19, Santa cruz	Mutivariable	Reported OS:6.4 (1.31–31.41)	3–120	7
Dhakal HP [30]	2012	468	Norway	IHC	Score ≥ 4 (292)	Clone SP-21, Thermo Fisher	NA	NA	NA	8
Kargi A [31]	2013	88	Turkey	IHC	> 10% (41)	Clone 4H-12, Abcam	NA	NA	74.2(1.9–93.7)	6

HR: hazard ratio; CI: confidence interval; NOS: New-castle-Ottawa; IHC: immunohistochemistry; TMA: tissue microarray; NA: not available.

**Table 3:** The assessment of the risk of bias in each enrolled study using the newcastle–Ottawa scale (NOS)

Study [ref]	Selection (0–4)			Comparability (0–2)			Outcome (0–3)			Total scale (NOS)
	REC	SNEC	AE	DO	SC	AF	AO	FU	AFU	
Costa C [11]	1	0	1	1	1	1	1	0	1	7
Ristimaki A [12]	1	1	1	0	1	1	0	0	0	5
Denkert C [13]	1	1	1	1	1	1	1	0	1	8
Spizzo G [14]	1	1	1	1	0	1	1	1	1	8
Wulfing P [15]	1	1	1	1	0	0	1	1	1	7
John K [16]	1	0	1	1	1	1	0	1	1	7
Surowiak P [17]	1	0	1	1	1	1	1	1	1	8
Gunnarsson C [18]	1	1	1	0	1	1	0	0	0	5
Park K [19]	1	1	1	1	1	0	1	0	1	7
Narssar A [20]	1	1	1	0	1	1	0	0	0	5
Zerkowski MP [21]	1	1	1	1	1	1	0	1	1	8
Zhang XH [22]	1	1	1	0	1	1	0	0	0	5
Glynn SA [23]	1	1	1	1	1	0	1	1	1	8
Miglietta A [24]	1	1	1	0	1	1	0	0	0	5
Rozenowicz RD [25]	1	1	1	0	1	1	0	0	0	5
Barisik NO [26]	1	1	1	0	1	1	0	0	0	5
Ciris IM [27]	1	1	1	0	1	1	0	0	0	5
Holmes MD [28]	1	1	1	1	1	0	1	1	1	8
Sondes KC [29]	1	0	1	1	1	1	0	1	1	7
Dhakal HP [30]	1	1	1	0	1	1	0	0	0	5
Kargi A [31]	1	1	1	0	1	1	0	0	1	6

REC: representativeness of the exposed cohort; SNEC: selection of the nonexposed cohort; AE: ascertainment of exposure; DO: demonstration that outcome of interest was not present at start of study; SC: study controls for age, sex; AF: study controls for any additional factors (chemoradiotherapy, curative resection); AO: assessment of outcome; FU: follow-up long enough (36 Months) for outcomes to occur; AFU: adequacy of follow-up of cohorts (≥ 90%). “1” means that the study is satisfied the item and “0” means the opposite situation.

### Association of COX-2 overexpression with clinicopathological features

**Figure 2:**
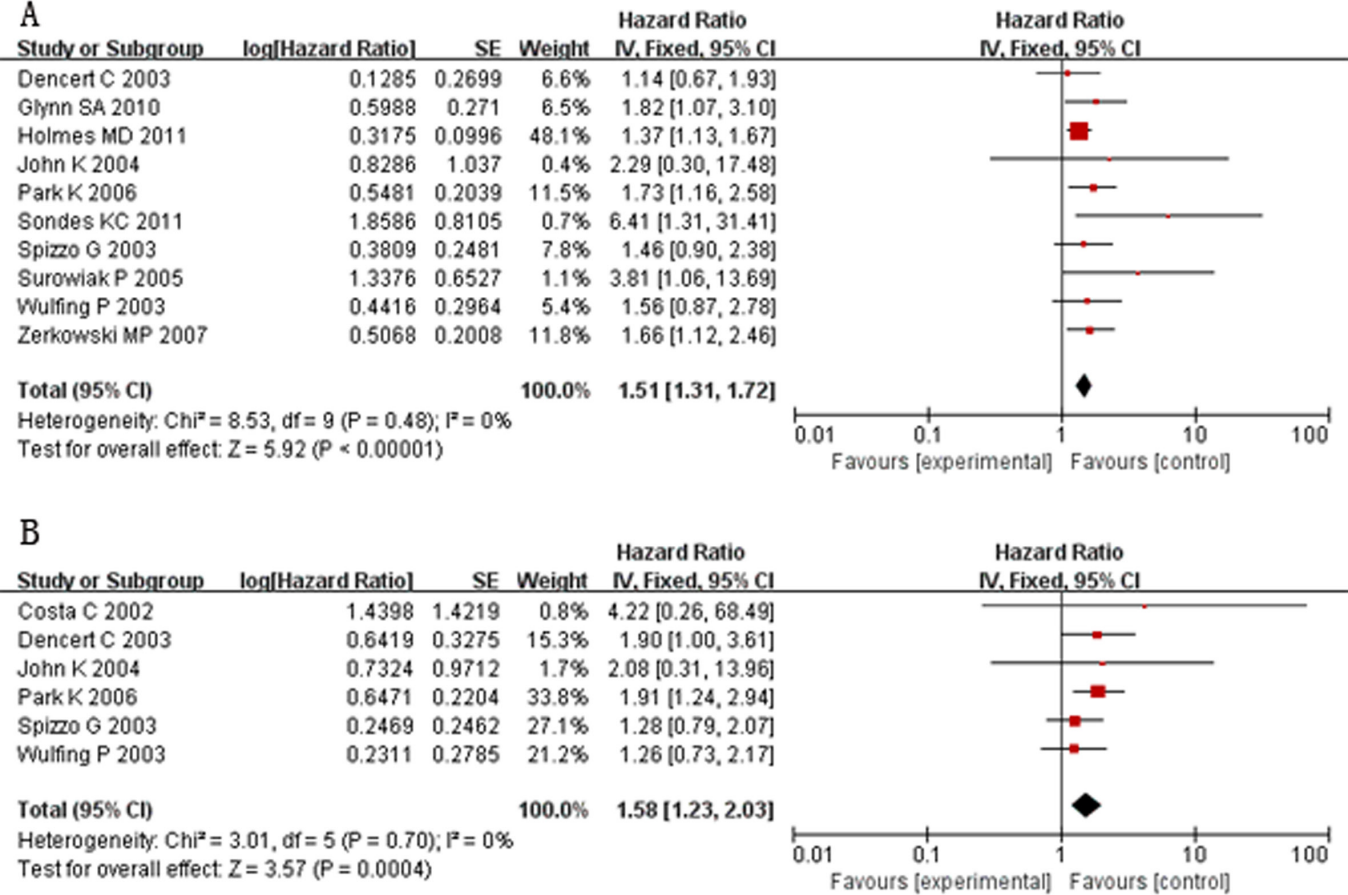
Forest plots of studies evaluating hazard ratios (HRs) of COX-2 for overall survival (**A**) and disease-free survival (**B**) with fixed effect model.

The relationship between COX-2 positivity and tumor size, lymph node metastasis, ER status, PR status, HER2 status, and vascular invasion status was considered in our meta-analysis. The pooled ORs using random-effect model were 1.71 (95% CI: 1.22–2.39, *I^2^* = 56%; *P* = 0.03), 1.76 (95% CI: 1.30–2.39, *I^2^* = 66%; *P* = 0.0004), 1.37 (95% CI:0.83–2.28, *I^2^* = 87%; *P* < 0.00001), 1.50 (95% CI: 0.85–2.63, *I^2^* = 87%; *P* < 0.00001), 1.49 (95% CI: 0.97–2.30, *I^2^* = 69%; *P* = 0.0004), and 1.57 (95% CI: 0.88–2.80, *I^2^* = 56%; *P* = 0.06) respectively (Figure 2). We found that increased COX-2 expression was significantly correlated with positive lymph node metastasis and bigger tumor size but not with ER status, PR status, HER2 status and the vascular invasion of breast carcinoma. In order to detect the source of heterogeneity among studies, we conducted “metareg” command using variables such as publication date, country, antibody catalog and detection method. The results showed that no variable included in the meta regression contributed to the heterogeneity.

### Association of COX-2 overexpression with survival outcome

**Figure 3:**
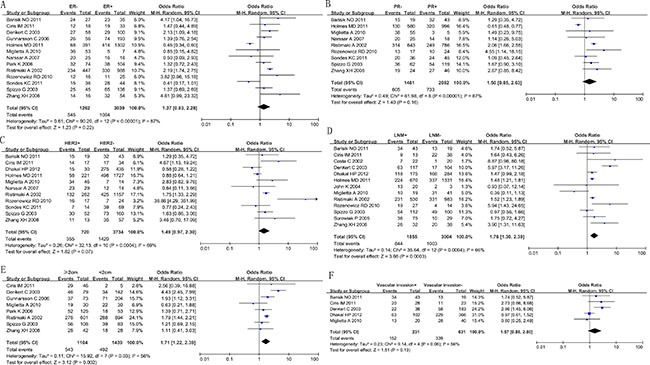
Forest plots of studies evaluating the association between COX-2 and clinical parameters in breast cancer with random effect model (**A**): ER status (negative vs. positive); (**B**): PR status (negative vs. positive); (**C**): HER-2 status (positive vs. negative); (**D**): lymph node metastasis (present vs. absent); (**E**): tumor size(≥ 2 cm vs. < 2 cm); (**F**): vascular invasion (present vs. absent).

Ten studies evaluated the relationship between COX-2 overexpression and OS of BC patients. The pooled HR with fixed effect model was 1.51 (95% CI: 1.31–1.72; *I^2^* = 0%; *P* = 0.48) (Figure 3), indicating high COX-2 expression significantly predicts poor OS of patients with breast cancer. To explore the heterogeneity with regard to OS, we performed subgroup analysis according to detection method, antibody catalog and analysis method (Table 2). Regarding diverse detection methods, subgroup analyses using a fixed effect model showed that increased COX-2 predicted an unfavorable prognosis by IHC (HR:1.60, 95% CI:1.21–2.13, *P* = 0.24) and TMA method (HR:1.48,95% CI: 1.27–1.72, *P* = 0.68). When subgroup analyses were stratified by the statistical analysis methodology, our results demonstrated that higher COX-2 expression was significantly correlated with poor OS both by univariable analysis (HR: 1.63, 95% CI: 1.14–2.31, *P* = 0.57) and multivariable analysis (HR: 1.48, 95% CI: 1.28–1.71, *P* = 0.28). Considering different anti-COX-2 monoclonal antibodies, COX-2 overexpression was predictive of worse OS for the studies applying clone 160112 (HR: 1.57, 95% CI: 1.28–1.93, *P* = 0.60) and other antibodies (HR: 1.45, 95% CI: 1.21–1.73, *P* = 0.20). It indicated that no variable mentioned above contributed to the heterogeneity in the results.

**Table 2:** Meta analysis results

Outcome	Variables	Number of studies	Number of patients	HR_FEM_ (95% CI_FEM_)	*P*_FEM_
**OS**	**ALL**	10	3919	1.51 (1.31–1.72)	0.48
	**Detection method**				
	IHC	6	879	1.60 (1.21–2.13)	0.24
	TMA	4	3040	1.48 (1.27–1.72)	0.68
	**Analysis method**				
	Univariable	4	529	1.63 (1.14–2.31)	0.57
	Mutivariable	6	3390	1.48 (1.28–1.71)	0.28
	**Antibody-catalog**				
	Clone 160112	6	1574	1.57 (1.28–1.93)	0.60
	Others	4	2345	1.45 (1.21–1.73)	0.20

HR: hazard ratio; CI: confidence interval; IHC: immunohistochemistry; TMA: tissue microarray; OS: overall survival; FEM: fixed effect model.

A total of six studies assessed COX-2 immunoexpression and correlated it to DFS. The combined HR with fixed effect model was 1.58 (95% CI: 1.23–2.03) given the absence of heterogeneity. Similarly, the overexpression of COX-2 was also significantly associated with poor DFS in breast cancer. Due to limited studies, no subgroup analysis regarding DFS was identified in the meta analysis.

### Publication bias and sensitivity analysis

**Figure 4:**
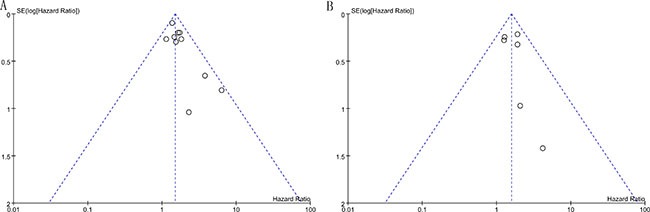
Funnel plots for all of the included studies reported with OS (**A**) and DFS (**B**).

Our statistical results showed that there was no evidence of publication bias in the funnel plot as it seemed to be symmetrical (Figure 4). Sensitivity analysis was performed on the eligible studies. The enrolled studies were sequentially omitted to investigate whether any single study could have an influence on the pooled OS or DFS. The results showed that the stable overall HR was found to be not dominantly influenced by each individual study.

## DISCUSSION

Since the 1980s, accumulating studies have showed that long-term treatment with non-steroidal anti-inflammatory drugs (NSAIDs) may lower the incidence of breast cancer development [32]. In vitro cell line researches suggest that NSAIDs and selective COX-2 inhibitors impede breast cancer cell growth [33–35]. Numerous *in vivo* animal experiments clearly indicate that high COX-2 expression is correlated to the genesis of mammary tumors that are sensitive to treatment with non-selective and selective COX-2 inhibitors [36, 37]. Recently, combining specific COX-2 inhibitors with conventional chemotherapy as a novel approach brings about some promising results in the field of BC treatment [38, 39]. Although the therapeutic effect of COX-2 is generally accepeted, evidence about the prognostic role of COX-2 in breast cancer is limited. As far as we know, this present meta-analysis is the first study to systematically assess the relationship between COX-2 overexpression and clinicopathological features/prognoses in BC patients.

The present meta-analysis of 21 clinical studies, which detected the COX-2 expression in BC tissue samples, indicated that elevated COX-2 expression was significantly associated with decreased 5-year OS and DFS rates of patients with mammary tumor. Additionally, when the clinicopathologic features were considered, the combined odds ratio(OR) was found to be significantly associated with bigger tumor size and positive lymph node metastasis of breast carcinoma. Based on these results, COX-2 might function as a valuable prognostic biomarker of breast carcinoma and provide a rationale for antitumor therapy on BC patients in future clinical trials.

Although our results showed that COX-2 acted to drive tumor growth and axillary node metastases of breast cancer, the mechanisms responsible for the above association remained unclear. There is some evidence that COX-2 is involved in the synthesis of prostaglandins, such as PGE1, PGE2 and PGI2 [40]. Interestingly, COX-2 and its products participate in the proliferation of mammary epithelial cells as they affect the synthesis process of DNA. Thus, the suppression of COX-2 with selective COX-2 inhibitors in breast cancer cells causes cell cycle G1 arrest and reduces the number of cells in S and G2/M phase, thereby inhibiting cell mitosis [41, 42]. Recently, new perspectives on COX-2 promoting tumor growth showed that the proliferation signals of BC cells were stimulated by estrogen acting upon the estrogen receptor(ER). ER induces c-Myb expression which in turn may stimulate COX-2, making estrogen more readily available in the cell. This positive feedback further promotes tumor growth. In addition, the molecular mechanisms underlying the impact of COX-2 on BC metastasis to regional lymph node remain largely unknown. The present studies indicate that COX-2 mediates VEGF-C expression depending on the endogenous PGE2 pathway regulated by the EP1/EP4 receptors, which may contribute to tumor lymphangiogenesis and lymph node metastasis [22, 43]. All the same, more studies are required to analyze the specific molecular mechanism of COX-2 overexpression facilitating breast cancer growth and metastasis.

Although our results are promising, our meta analysis has several limitations. Firstly, the sample size of most enrolled studies was relatively small. Secondly, the high variability for COX-2 protein expression reported by different authors could partly be attributed to the inconsistent scoring methods, protocol of staining and cut-off points for COX-2 immunoexpression. Thirdly, few studies explored the COX-2 expression in Asian population, which might bring out a certain publication bias. Fourthly, most of the enrolled studies were retrospective studies rather than randomized prospective studies.

In conclusion, the present meta-analysis suggest that COX-2 up-regulation can predict an unfavorable prognosis of BC patients. Our results also indicate an association of COX-2 overexpression with clinicopathological features such as bigger tumor size and lymph node metastasis. More multicentre and prospective studies are needed to clarify the clinical relevance and precise molecular explanation for the abnormal expression of COX-2.

## MATERIALS AND METHODS

### Search strategy and study selection

The electronic databases PubMed, EMBASE, Web of Science, the Ovid Database and Grey literature were searched for studies to include in this meta-analysis up to May 31st, 2016. The key words were searched as follows: “breast cancer” or “breast carcinoma” or “mammary gland cancer” or “breast tumor” or “breast tumour” or “breast neoplasm”, “COX-2” or “Cyclooxygenase-2”, and ‘‘prognosis’’ or “survival” or “outcome”.

To be eligible for inclusion in this meta-analysis, a study must meet the following criteria: (1) the correlation between COX-2 expression with BC patients’ survival (ie, overall survival [OS] and/or disease free survival [DFS]) was investigated; (2) the expression of COX-2 was measured by immunohistochemistry (IHC)/Tissue microarray(TMA) method in the primary BC specimen; (3) the correlation between COX-2 and clinicopathological features of breast cancer was described; (4) all selected BC patients were pathologically confirmed; and (5) the median follow-up period was no less than 24 months. All candidate manuscripts were carefully read by two independent authors (XF and JHC). To reach a consensus, disagreements on conflicting results were resolved between the two authors.

The exclusion criteria were as follows: (1) non-English articles; (2) non-human studies; (3) case reports, review articles, or letters; (4) duplicate publication; (5) with no more than 20 eligible BC patients; and (6) with insufficient data to calculate the hazard ratios(HR) and its 95% confidence interval (95% CI), or the Kaplan-Meier curve in the article could not be extracted.

### Data extraction

All relevant articles included were screened and assessed independently by two investigators (XF and JHC). To identify high-quality studies, each publication was scored based on the New-castle-Ottawa (NOS) Quality Assessment Scale [44]. Study with a score of 6 or higher was considered as a high quality study. Information was carefully extracted from the full publications, including the following items: first author, number of patients, year of publication, country of origin, detection method, cut-off value, antibody for COX-2 staining, positive COX-2 expression, analysis method, hazard ratio (HR) for survival (OS and/or DFS), follow-up time, and quality assessment. To get the survival data that were not reported by the authors, we digitized and extracted the data from the Kaplan-Meier curves in the articles using the software designed by Jayne F Tierney and Matthew R Sydes [45].

### Statistical methods

According to the guidelines proposed by the Meta-Analysis of Observational Studies in Epidemiology group (MOOSE) [46], enrolled studies were divided into two groups for analysis: those with data regarding OS and DFS. Hazard ratios (HRs) and 95% confidence intervals (CIs) were used to combine as the effective value. For the pooled analysis of the correlation between COX-2 overexpression and clinicopathological features, odds ratios (ORs) and 95% CIs were combined to estimate the effect. *I*^2^ and *Q* tests were performed to calculate the heterogeneity of the individual HRs/ORs. A probability value of *P* < 0.1 and *I*^2^ ≥ 50% indicated the existence of significant heterogeneity. A fixed or random effect model was used depending on the heterogeneity analysis. For these analyses, *P* < 0.05 was considered to indicate significance. Publication bias was assessed using Begg's funnel plot and Egger's test. All of the calculations were performed by Review Manager version 5.3 (Cochrane Collaboration, Oxford, England).
